# Global Agri-Food Sector: Challenges and Opportunities in COVID-19 Pandemic

**DOI:** 10.3389/fsoc.2021.647337

**Published:** 2021-07-12

**Authors:** Saima Hamid, Mohammad Yaseen Mir

**Affiliations:** ^1^ Centre of Research for Development, University of Kashmir, Srinagar, India; ^2^ Department of Environmental Science, University of Kashmir, Srinagar, India

**Keywords:** global pandemics, agricultural commodities, world economy, routine demands, under developed nations, food security

## Abstract

COVID-19 pandemic has been catastrophic for almost everything including the global economy. Among many sectors, the food and the agriculture sector was the worst hit following the immediate lockdown and market shutdowns. Though some stability was prevalent from supply side till date, however, the severe restrictions put in place to curb the spread of pandemic have endangered the supply of agricultural and food articles contemporaneously across borders and from field to fork. While the income decline due to price falland supplies chain disruptions due to pandemic have escalated the food shortages in several of developing and developed countries. Nevertheless the global demand for food items has remained more or less unchanged owing to their inelastic demand. Even within the global level, the scenario of food security and supply chain stability has been substantially deplorable for emerging and less developing countries due to their lack of insulation to the global shocks or pandemics. Notably, the technological backwardness, excessive know-how dependence and denied accessibility on several grounds lead to poverty and food hunger in these countries. At the policy level, a holistic approach specifically targeted towards the developing and less developed economies is highly warranted to ensure an appreciable progress towards the minimisation of sensitivity with regard to agriculture and food security. Apart from the measures to insulate them from global shocks, additional steps need to be taken to alleviate their technological backwardness and denied accessibility on certain socio-cultural norms.

## Introduction

SARS-nCoV-2 is a novel virus known to cause COVID-19 disease which is responsible to 1.6 million deaths in six continents of the globe as World Health Organization (WHO) declared state of health emergency on March 11, 2020 due to this pandemic disease and so far global total of cases 71.6 million are confirmed till date (WHO, 2020a; Hamid et al., 2020). WHO (2020a) issued Strategic preparedness and response plan to implement the measures regarding community participation, temporary travel restrictions, social gatherings, closure of educational institutes and work places. Work from home recommended for various sectors but it cannot be applied to food sector that needs to work in their daily routine. With regards to the economy, the food industry is a very significant field which is life sustaining than the rest of sectors like as tourism and aviation after a pandemic, the food industry faces various sets of problems. The pandemic could lead to an aviation loss of US$113 billion and a tourism industry loss of US$80 billion (IATA, 2020; FAO and WHO, 2020). Preserving the welfare of the employee and having enough staff instead of those who do not choose to work because of pandemic remained big concern shared by all food corporations. In order to keep food chain alive it was mandatory for the management to supply and distribute food and to work on continuous manner during pandemics. With the contribution of all parties, the management of the distribution of food and services across the supply chain should be assured. Ensuring customer interest is also important for food quality and protection (UNTWO). At this moment of crisis, food sustainability is related to the proximity of customers to food rather than access to food (OECD, 2020d). No study reveals that COVID-19 has to date been spread through food intake, in view of the large size of the pandemic. However in Xinfandi market, new infections have been seen due to processing of salmon fishes which can be inferred that the risk of the virus that spread by foods is lower than the perceived risk. SARS-CoV-2 can be dangerous source of food borne transmission while taking consideration of its survival in a number of environments, such as rubber, steel or cardboard, animal tissue (meat, fish or poultry). Food business operators’ hygiene controls are intended to avoid food contamination by any pathogen and would also aim to prevent food contamination by the COVID-19 virus (European Commission, 2020a; FAO, 2020b). Any cooking and eating habits, however may contribute to the reappearance of corona virus from animals to humans (Pressman et al., 2020).

### Effects of Pandemic on Food Supply Chain

Agricultural production, postharvest handling, processing, distribution/retail/service, and consumption i.e., field to fork are the 5 phases of Food supply chain (FSC). In the food supply chain, two mechanisms surrounding food consistency and protection are used. The first is focused on rules and legislation that use compulsory requirements that are reviewed by state departments. The second is focused on voluntary principles established by business laws or international organisations (Rizou et al., 2020). According to Rizou et al., (Pressman et al., 2020), FSC involves critical last stages where people can get infected easily, hence for the safe handling/preparation/delivery of food, using personal protective equipments such as helmets and glove, sanitization of surfaces and working environments, even the maintenance of social distance are some Safety measures to ensure the continuity of food flow. The COVID-19 pandemic does not specifically impact development, unlike foot and mouth disease, bird flu or Listeria, since it does not propagate directly to animals or agricultural products (Richards and Rickard, 2020). However as a result of the pandemic, policymakers around the world have placed major limits on the flow of goods (land, sea and air transport) as well as on labor mobility. Reports have indicated that the use of food delivery vehicles has reduced to 60% after the constraints in France were 30% before the pandemic (IATA, 2020; OECD, 2020c). Temporary or seasonal sort of employment is common in developing and underdeveloped countries, particularly when planting, sorting, harvesting, refining, or transporting crops to markets. Therefore, due to the lack of local or temporary workers due to illness or travel restrictions enforced by the lockdown, the supply chain is greatly impacted. In situations where the illness specifically impacts their health or activity, it also weakens not only the processing ability of others but also their own food protection (ILO, 2020a). The lack of labor due to the pandemic crisis has led to significant disturbances in certain industries, such as livestock production, horticulture, planting, harvesting and crop processing, which are relatively labor intensive. Farm worker shortages, however, were already a significant concern long before the COVID-19 epidemic (ILO, 2020b). The “Pick for Britain” campaign in Britain was planned to locate 70,000 British working in the field and through the harvest (Deng et al., 2015). However, owing to the lack of labor due to sickness and the physical distance to be sustained during production, the crisis is weakening the opportunity to work for farms and agricultural undertakings. These conditions delayed the delivery of grain and agricultural inputs and produced difficulties with the continued supply of food to markets (Author Anonymous, 2020b).

### Effects of Pandemic on Global Food Trade

While the current circumstances appear unprecedented, even before the COVID-19 crisis, food supplies were vulnerable to climate-related and disease-related issues. Food markets have historically been fragile due to numerous incidents and shocks, such as the oil crisis in the 1970s, the outbreaks of SARS and Ebola, and the food crisis from 2006 to 2008. Only a year ago, Africa’s Swine Fever outbreak upset the world commodity markets and became a progressive epidemic in Eastern Europe and Asia. By the end of 2019, China, the world’s biggest pig manufacturer (1/3 of the global market) and largest exporter had lost 37% of its pigs (IPES, 2020). In certain African countries, the production, marketing, and trade economies regarding agriculture where Ebola created huge damages. The ongoing COVID-19 crisis has modified certain governments’ food trading policies, aimed at limiting exports and making imports simpler. Ensuring the preservation of the number of goods in the domestic market is the key reason why countries implement export restrictions. Although this outcome is usually produced by an export limitation in the short term, it still has some negative consequences. Ban on export resulted domestic price drop due to which farmers economy got hit *via* low crop production and decreased incentives in the industry. As well as export controls lead to a reduction in domestic markets, triggering a financial downturn to producers and reducing business incentives. Secondly, by losing their position on foreign markets, countries would lose their economic edge. The third explanation is that export controls damage the image of the exporter and allow importers to decrease confidence in the global markets, thus reducing foreign trading trust and undermining potential export business prospects (Espitia et al., 2020).

### Impact on Food Production and Distribution

In order to monitor the rate of infection, most nations have taken steps such as home confinement, travel restrictions and business closure. Such regulations have a huge effect on the food delivery at any point of the food supply chain. It is estimated that world trade in goods will decrease from COVID-19 by 13–22% (FAO, 2005). Different areas of agriculture have received serious pandemics, such as wheat, livestock and fisheries. With inadequate access to animal feed and a lack of work, (WTO, 2020), COVID-19 in China has had a greater effect in livestock production. Travel ban has limited the availability of reproductive supplies of poultry in many countries. Prolonged restrictions on travel vanished the breeding stock and hatching eggs as per reports of The International Poultry Council (IPC) (Zhang, 2020). As we know the cheap source of protein for 3 million people thus accounting more than 20% of animal protein for the human consumption (Vorotnikov, 2020). In various parts of Asia, Africa and Europe, aquaculture suffered huge losses due to labor shortage, inadequate input supplies when the other main causes were social distance and lack of feed (FAO, 2020e). Farmers are required to store their unsold produce for a longer period of time, which leads to a decrease in food quality as well as a rise in production costs (FAO, 2020e). COVID-19 has been struck worst by the supply of milk and dairy products. After a substantial decline in milk production and the closing of the milk manufacturing business, dairy farmers are forced to dump milk and milk products. Dairy producers in America report that nearly 4 million gallons of milk were spilled every day by farmers nationwide (Forstadt, 2020). In Nepal, 2 billion NPR dairy products have been destroyed and 5 billion NPR dairy products in storage tend to be on the edge of deterioration (NepaliSansar, 2020). Since the Great Recession of 1929 to current pandemic of covd-19, world is suffering from huge loss in terms of economy, social and psychological which set the countries on worst crizes according to International Monetary Fund (Marlow, 2020) among the past epidemics including the 2002–2003 SARS (Severe Acute Respiratory Syndrome) epidemic; the 2003 North American BSE (Bovine Spongiform Encephalopathy) crisis and the 2003–2004H5N1 avian influenza epidemic. Compared to 2019, a 4.9% decline in global Gross domestic product (GDP) has been projected for 2020; the European Union (10.2%, with peaks for Italy and Spain, both 12.8%, and France, 12.5%), the United Kingdom (10.2%), Canada (8.4%), and the United States are among the most significant economies experiencing losses (8.0%). In spite of the fact that the corona virus is extremely infectious and that an antidote is not yet available on the market, the forced shutdown of industrial and business operations has created chaos in the entire economic sector (Cucinotta and Vanelli, 2020). The pandemic will shock the supply and demand sides of market demand, many economists say. The former applies to interruptions in the provision of goods and services, while the latter refers to the volume of consumption and procurement of products (OECD, 2020a). Standard food supply chain (FSC) functions are impeded by COVID-19 including farmers, manufacturing plants, wholesalers, and retailers (ILO, 2020a) form a diverse FSC. Breakdowns or bottlenecks in some section of the FSC have impacted other components up and down the chain during the current COVID-19 pandemic. The findings of recent analyses have shown that the shock in the supply of labor has undergone the largest decrease (OECD, 2020a; Johnso and Mue lle, 2002), leading to instability of the supply chain and dumping or waste of foodstuffs at fields. This instability is due to the absence of grain harvesting, the aggregation of farm goods, and the interruption of the distribution network (Cucinotta and Vanelli, 2020). The shelves of grocery stores (supermarkets) were often vacant as a result of this FSC disturbance (a lack of workforce in packaging and selling goods to retailers), which was also attributed to hoarding and panic buying, which in turn contributed to the scarcity of essential foodstuffs (NepaliSansar, 2020). Many policymakers have reduced the selling and export of foodstuffs and boosted imports of essential products (CDC, 2020a) to avoid such a shortage. The lack of supply in retail stores and the growing demand from households have had a substantial effect on the volatility of agricultural product prices (Cucinotta and Vanelli, 2020). In the meantime, a massive demand shock has been reported in the hotel, restaurant, and catering (Ho.Re.Ca.) sectors, with a big effect on the food system (CDC, 2020b; WHO. HIV/AIDS, 2020). The global pandemic expansion has and will continue to have an unparalleled detrimental effect on households and firms’ existing and future livelihoods. Consumer conduct, as a buying decision mechanism, is a behavioral process, as described by Engel et al. (Lopez-Ridaura et al., 2019), which is observed before and after purchase. The action of consumers is very dynamic, requiring a wide variety of activities, from intake to disposal (Zavatta, 2014). Several influences including global, geographic, social and demographic diversity, as well as consumer tastes and attitudes, which are all of feed intake (World Bank, 2019).

### Agro-Food Consumption Habits and Preferences

**FIGURE 1:**
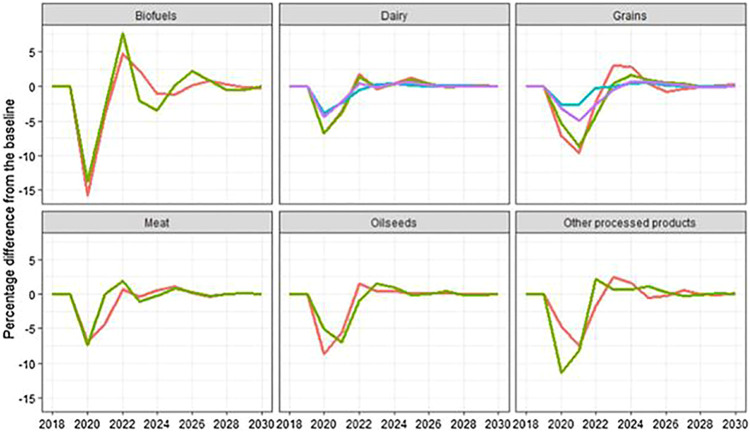
Impacts on world prices on the basis of Percentage difference from the baseline [Source: Elleby et al. (2020)].

Since the FSC and food supply were affected by the economic crisis and the occurrence of the COVID-19 pandemic, many have resolved this deviation from normality by changing their food preference reactions and behavior. Bree (2020) has indicated that forming a new habit typically takes approximately 3 weeks to develop. Clearly, the COVID-19 crisis lasted well longer than 3 weeks, but what began as a transition in customer behavior has now become a habit. According to the EY Future Consumer Index by Rogers and Cosgrove (2020), 28% of cautiously lavish consumers (25% of the 4,859 consumers surveyed in the United States, Canada, the United Kingdom, France and Germany during the week beginning April 6, 2020) will change their eating habits as they change their eating habits, according to the EY Future Consumer Index by Rogers and Cosgrove (Hubbub, 2020) of the five consumer segments to take on prominence as the COVID-19 crisis can be said to have ended. Since after lockdown implementation from 16th March 2020 in United Kingdom changed the cooking and eating behavior among 90% of a representative sample of 2,000 adults surveyed as per research conducted by Hubbub (Datassential, 2020). People remained indoors and spend long hours to prepare meals while enjoying cooking at home (44%); and “sharing” virtual meals over Zoom, Skype, Facetime etc., (40%) and with neighbors (47%). Such recently discovered dietary patterns include better menu preparation, the use of cupboard staples, the freezing of food/meals and the increased use of leftovers. As per research conducted by Hubbub (Allen, 2004) also found that many individuals did not eat as much fresh fruit and vegetables as normal (31%), reducing their interaction with shops, while some also decreased their milk/egg consumption throughout the lockdown era (15%) (Figure 1). Shortages and challenges in obtaining staple food ingredients caused many to attempt new recipes (22%). Finally, there are signs that these emerging habits will persist after the limits have been greatly removed, albeit to a lesser degree (Table 1) (Elleby et al., 2020).

**TABLE 1:** Shows the estimated impacts on global food prices until 2025 on comparison with GDP baseline shocks.

Items	2020	2021	2022	2023	2024	2025	Band (%)
Biodiesel	−15.9	−4.0	4.7	2.3	−1.0	−1.2	22.4
Butter	−6.8	−3.6	−1.7	−0.3	0.4	1.3	4.6
Skim milk powder	−3.8	−2.4	−0.4	0.3	0.5	0.2	0.9
Rice	−2.7	−2.7	−0.3	0.0	0.4	0.5	2.7
Wheat	−3.2	−4.9	−2.6	−0.4	0.7	0.7	3.9
Beef and veal	−10.4	−0.5	6.9	−1.5	−3.0	0.9	1.8
Vegetable oils	−11.4	−8.1	2.2	0.7	0.7	1.1	6.6
Poultry	−7.0	−4.2	0.7	−0.3	0.5	1.1	3.2
Pork	−17.6	4.7	7.2	−4.3	−0.8	2.9	3.7
Total protein meal	−4.7	−7.5	−1.7	2.5	1.6	−0.5	4.5

### Global GDP and Pandemics

However, uncertainty remained as to how long the COVID-19 recession will last and what the medium-term global economic effects will be. It relies on many factors, including agricultural commodities that affect the supply and demand. These include how fast multinational companies will resort to lock-outs; whether secondary waves will cause policymakers to implement new lock-down measures; how quickly the SARS-CoV-2 virus can be vaccinated and/or successful treated, and how any of it affects market habits. However the COVID-19 effect on their GDP estimates already represents a variety of global economic outlooks. The projections for the global GDP reduction in 2020 by the IMF, World Bank and OECD range from 3.0 to 7.5% and the forecasts for the resulting global GDP rise in 2021 range from 2.8 to 58%. (IMF, 2020; World Bank, 2020a). The International Food Policy Research Institute (IFPRI) forecasts that the economic downturn in 2020 will increase the number of people living in severe poverty by a whopping 20% or 140 million people, resulting in expanded food insecurity in many countries, building on the IMF projection (Laborde et al., 2020). In countries dependent on seasonal migrant workers in the agro-food industry, a sudden loss of mobility across borders and within countries has triggered labor shortages, which in turn has impacted food supply and prices globally (Hernandez et al., 2020). For example, the prices of some main staples in India and in several African countries have reportedly risen by more than 15% from pre-COVID-19 levels (OECD, 2015). The pandemic has also influenced trade in goods by, for example additional border controls, lack of shipments of freight and improved sanitary controls. In addition, the pandemic, analogous to the food crisis of 2007–2008, caused several countries to enact export bans in order to protect their domestic customers (Author Anonymous, 2020a). These trade frictions may impact global food prices as well. The Organization for Economic Co-operation and Development (OECD) Secretariat and the Food and Agriculture Organization of the United Nations (FAO) developed recursive-dynamic partial equilibrium model namely Aglink-Cosimo which is an outcome of their collaboration (Araujo-Enciso et al., 2015; EC., 2019). In order to project the baseline for the main agricultural commodities over the medium term, this modeling approach has been used to produce the OECD-FAO and EU Medium Term Agricultural Outlooks (FAO., 2020f). A single scenario, augmented by EU from the spring 2020 Economic Prediction by the European Commission, is evaluated based on country-specific GDP growth projections in the IMF, World Economic Outlook database (April 2020). The scenario shocks are the GDP growth rates expected by the 2020 and 2021, the GDP base line and the 2021 scenario. We believe the GDPs come back to their core principles from 2022 and beyond.

### Global Impacts

A stochastic theoretical study has been conducted on the relationship between foreign oil prices and the agricultural product markets in addition to the scenario impacts, expressed as a range of point’s forecasts. The agriculture and energy sectors are interlinked primarily through the output of bio-fuels (mainly mandated) and the cost of input (e.g., fertiliser costs). At present, due to a mixture of supply and demand factors, we are facing a time of low oil prices. As discussed below, however, foreign oil prices in the model are exogenous and we have not made any conclusions regarding their divergence from the baseline to retain a strict emphasis on the impact of COVID-19 on production. Instead, based on the historical volatility of oil prices, we calculate the joint distribution of scenario effects, where the variation derives from alternate oil price projections. A declining trend has been observed for the prices of vegetable oils, meats and bio-fuels as well as same trend were found for agricultural commodities in 2020. On comparison with baseline data, the prices will be underneath in 2021 for some lamb and pork flesh. The illustration is more mixed for 2020, with the grains and bio-fuels above and below the baseline. All product prices are near and close to baseline values as we come to 2025. Until the end of the 2030 prediction era, this will continue to happen. Mainly through production of bio-fuels (managed to a significant degree) and input prices, agricultural and energy markets interconnect (e.g., fertiliser costs). At present, due to combined supply and demand considerations, we are faced with a time of low price. Trade ties international economies with the global economy. As a result, a rise in inflation on the global economy is generally often responsible for an increase in internal prices.

### Recommendations to Minimize the Effect of Covid-19

The outbreak of the COVID-19 severely endangers food security, nutrition and welfare. The financial chaos caused by the pandemic risks the access to food economically and physically accessible. Disrupted marketing, logistics and commercial networks, and potential problems could limit access to food in some parts of the world (FAO, 2020c). World Food Program study has indicated that by 2020, COVID-19 will increase the number of individuals suffering serious poverty to 265 million (WFP, 2020a). Another research undertaken by Headey et al. (WTF, 2020a) found that COVID-19 contributes to a rise of 14.3% in the incidence of lack of health and social security for low or middle-income children under the age of 5 years of age.

### Actions on Global Trade

It is important to continue the movement of agricultural inputs between countries, even in the case of quarantine restrictions or the closure of borders. Acts should also be taken in the short term to encourage trade in agricultural inputs such as machinery and fertilisers, as these needs are essential for the smooth continuity of planting activities (Headey et al., 2020). Trade and tax practises need to be discussed to keep free trade open. At the beginning of the COVID-19 epidemic, some of the big exporting countries adopted the “beggar thy neighbor” approach that requires importer countries to cover the costs or dangers of insufficient supply. The distributional consequences of “beggar thy neighbor” often include food price spikes and a reduction in food security (FAO, 2020d). Countries should also lift export prohibitions and import taxes because the food prices can be avoided by lowering import tariffs due to low food supply (Headey et al., 2020). As a result, the protectionism of food trading included various types of taxes, tariffs, non-tariff barriers and restrictions (Barichello, 2020). However the introduction of these policies has led to a disparity between demand and supply, contributing, in the medium and long term, to a sharp increase in global food prices. Therefore the most disadvantaged group of the remaining players in the supply chain is the economically marginalized clients.

### Sociological Theories for Food Security

At the end of the Cold War and a shaky global surge in democracy, a flood of new information technologies that bring the global community closer together and contribute to the rapid expansion of globalization, the explosion of a global HIV/AIDS pandemic, and a 35% increase in the world’s population have all been witnessed by the world. Simultaneously, there are constants like violence and war, widespread poverty and inequality, and ongoing environmental challenges. Hunger persists in all of this, and sociology’s role in solving it requires further and more attention. As per the reports of FAO, (Food and Agriculture Organization of the United Nations, 2008), and (FAO, 2021), Less developed Nations are the hotspot which comprises of nearly 96% of the world’s total hunger population as these 82 nations have been categorized “low-income food deficit” countries with chronically poor, net importers of food and are prone to diseases including covid-19 while the Hotspot for hunger lies in Sub-Saharan Africa and South and Southeast Asia. Children under age five, who include the huge mass of the world’s food insecure, encompass 18,000 of the 25,000 people per day who die of hunger, adding together more than 6.5 million per year [U.N. (World Food Programme)]. Food insecurity is defined by shortages, poverty, and suffering, according to DeRose et al., (1998). Food insecurity is most directly connected to inequality, with a focus on distribution and variables that affect food access. When people are unable “to secure sufficient food to satisfy the nutritional needs of their family members owing to insufficient income, limited access to productive resources, inability to benefit from private or governmental food transfers, or lack of other entitlements to food,” they are said to be in food poverty (Uvin and Yverdon, 1994). The present global food crisis due to covid-19 pandemic is a good illustration of how food insecurity may have far-reaching consequences particularly for the poor but also for people who appear to be food secure such as those in the middle class who feel the sting of rising food prices. Food sovereignty, according to McMichael (2004), is “a community’s or country’s social right to set its own policies surrounding food security (enough supply and acceptable cuisine) and the cultural, social, and ecological circumstances under which it is sustained” (Menezes, 2001). Food insecurity is as much a function of political economics and the global economic system as it is of population and technology. Food insecurity’s persistence underscores its worldwide relevance (Devereux, 2007). Unlike previous crizes, globalization has fostered interdependency, where issues in one part of the world influence difficulties in another. This is the setting that necessitates a new social understanding of food security/insecurity. As a result of globalization, governments have shifted from feeding themselves to exporting cash crops to the rest of the world. Buying food for consumption in the “global food order” and becoming net food importers on the market (Friedmann, 1982). In order to compete with global agri-business, local markets and prices are disrupted, and peasants who grow crops for local use are evicted off their land. Ironically, many of the people who create the world’s food supplies are hungry themselves (Barkin, 1982; McMichael, 1995). In India, a shortage of labor, storage, or transportation choices resulted in losses for 40% of farmers who faced a production drop in April 2020. Small and marginal farmers made up around 52% of the respondents, landless farmers made up 6.7%, medium farmers made up 19.9%, and large farmers made up 20.7%. Over half of the farmers claimed harvesting cost more this season than the previous season, either to a lack of labor or machinery, or a greater cost of machinery. Food security/insecurity is political in terms of its ties to social movements and social transformation, in addition to macro-structural, global political economy processes. Food and hunger-related collective action has a wide range of applications including food riots (McMichael, 2004) and food justice movements (Walton and David, 1994) in addition to sustainability (Allen, 2004; Wekerle, 2004), cooperative (Buttel, 1997), food sovereignty (David and Michael, 2004), and local/slow foods movements (Petrini and Gigi, 2006; Schnell, 2007). The freegan subculture, which gleans food that has been thrown away, including dumpster diving as a political act, draws attention to food waste and global consumption patterns by gleaning food that has been thrown away (Edwards and Mercer, 2007). When many people go hungry, freegans fight the unfairness of overconsumption and inequity. The act of eating may plainly be political, and sociology of power, politics, and social movements has a long history of helping to grasp its importance for global food security and insecurity. Food insecurity is linked to a variety of factors, including class, ethnicity, and gender as well as development, land availability, rural-urban inequities, and age. Food insecurity is mostly caused by a lack of financial resources to purchase food. The poor are the hardest hit among these persons, resulting in a situation in which the country performs significantly worse than its contemporaries in the industrialized world. This is especially true in light of the present economic slump, which has resulted in the formation of new and spreading “food deserts” in the United States, where people are either jobless or going hungry for the first time in their lives. According to Poppendieck (1995), insecurity of food generates a scenario of “heat or eat”. People forgo eating for rent, services or medical charges. In addition to the substantial US welfare reform in 1996, the importance of stratification for food insecurity becomes even more apparent, combining poverty with gender and ethnic discrimination. Moreover, despite their role in all stages of food production, distribution, and processing, food insecurity among women and girls in the globe continues to be pervasive. Extending this to racial disparity worldwide demonstrates how lamination systems pose as obstacles to food distribution and other fundamental necessities. Among other locations, state failures in Eritrea, Ethiopia, Indonesia, Somalia, Sri Lanka, and Sudan (Messer et al., 1998) demonstrate the plague of ethnic disparity for food security. Food security and insecurity addressing are crucial to international peacekeeping and security efforts in conflict areas (Bryant and Christina, 2005). Food security is a crucial component of how stable, sustainable societies are created with the strong links to poverty and underdevelopment.

### Applicability of Theory of Access to Food Security

As defined by the theory of Access distinguishes between one’s right to access resources and one’s ability to profit from them. People may have the right to access a resource, but due to a lack of structural and relational mechanisms such as capital, technology, labor, knowledge, authority, market mechanisms, social relations, and identity they may not be able to use the resource in a productive way to benefit from it (Ribot and Peluso, 2003). It has also been highlighted by Uvin (Devereux, 2007) that food insecurity includes numerous components. According to McKay and Colque (2016), accessing resources requires procedures that go beyond legal norms or titles, and that a lack of such processes leads to exclusion. Suppose a farmer could have the right to utilize the land but not the labor or cash to rent it. The most significant resource for agricultural productivity for smallholder farmers is land, followed by irrigation water. Water used for irrigation aids agricultural crop development and mitigates the impacts of insufficient rainfall. Access to productive resources may assist small farmers, through improving production and adapting to and mitigating the climatic changes, to implement sustainable land management measures, such as water conservation measures and nutrient management. Food security is one of the prerequisites or outcomes of a livelihood. Smallholder farmers are subject to food insecurity and have unsustainable livelihoods due to a lack of access to productive resources. The majority of population in the least Developed nations including India, Somalia, Kenya, Pakistan and other countries whose livelihood depends on Agriculture and livestock production. In order to check the wide applicability of Theory of Access, a small study has been carried out in north-western slopes of Mount Kenya, covering parts of Laikipia and Meru countries, as this theory holds broader significance while considering different variables (Swindale and Bilinsky, 2006). For households which depend on agricultural and livestock production for their livelihoods, access to production resources, such as land and water is essential. Research generally assumes that the tenure of resource security (expressed as a “bundle of property rights”) is favourable for agricultural output, and consequently food security.

However in the category of bundle of rights and powers, following variables which form state of art include Access to Right, technology, Markets, knowledge, labor and labor opportunities, capital, Access through social identity and through social relationships, Rights-based access to irrigation water. Household Dietary Diversity Score (World Food programme, 2008), Food Consumption Score (Maxwell and Caldwell, 2008), Coping Strategy Index (Coates et al., 2007), Household Food Insecurity Access Scale (Bilinsky and Swindale, 2010), and Months of Inadequate Food Provisioning (Mutea et al., 2020) were used to estimate the food security status of the tested homes. Mutea and co-workers in 2020 assessed in their study whether each family is satisfied with the food security requirements for each of the five indicators to get a sense of their overall food security situation. In order to categorize safe and insecure food, Mutea et al. (World Food Programme, 2020a) utilized the food safety thresholds for the respective indices. Household food security via the lens of the Theory of Access has an application that has yet to be fully explored for industrialized countries in order to determine the optimal relation for each variable. The majority of the farmers in this research had property rights to their agricultural resources and were able to profit from them. Instead, it indicated that the fundamental issue was a lack of access to the technology required to unlock additional advantages from households’ productive resources, leaving these households exposed to food insecurity. Hence, greater number of variables can be included even can compared for the Least developed Nations in order to frame the components which are lacking or have put the poor families or farmers livelihood into halt as making them prone to hunger and food security and also this theory can be used for comparative analysis in order to determine changing variables foe all the countries which comes under the category of food insecurity.

### Statistical Analysis Over 45 Developing Nations for Food Security

By the end of 2020, the number for acute hunger will double as per findings of United Nations World Food Program (WFP) (Food and Agriculture Organization of the United Nations et al., 2020). The pandemic COVID-19 will result in addition of 83–132 million people into the category of malnutrition by 2020 according to estimates of Food and Agriculture Organization (World Trade Organization, 2020). While adapting measures to abate transmission rates, The World Trade Organization (WTO) (Food and Agriculture Organization of the United Nations) reported that countries like Egypt, Thailand, North Macedonia, Ukraine and Kyrgyzstan started ban over certain food and agricultural products. Some countries which are largest suppliers of wheat like Russia, Rice like Vietnam implemented export-restrictions while majority of countries put forth custom restriction *via* cargo export as pandemic has escalated the tensions between the United States and China, in which food exchange taris have been used as an instrument of economic pressure intensively (Erokhin and Gao, 2020).

Erokhin and Gao (World Bank, 2020b) tried to understand the relation between food security, food trade, dynamics of COVID-19 cases, currency volatilities and food inflation by dividing the 45 developing nations into three group studies in order to carry out statistical analysis using Yamamoto’s causality test, variance decomposition, autoregressive distributed lag method on the basis of level of income. With a gross national income (GNI) per capita of $1,025 equal or less according to norms of World Bank (Puma et al., 2015) have kept under Group I likewise GNI per capita between $1,026 and $3,995 comprises Group II countries and GNI per capita between $3,996 and $12,375 includes Group III. This study pertains to check the dynamicity among different variables which were included as

1. Y = Number of people with insufficient food consumption (Unit-millions of people),
2. X1 = Number of confirmed COVID-19 cases (Unit-Number of cases),
3. X2 = Balance of food trade (USD million),
4. X3 = Food inflation (Percentage) and
5. X4 = Currency exchange (Unit-Monetary units)

**FIGURE 2:**
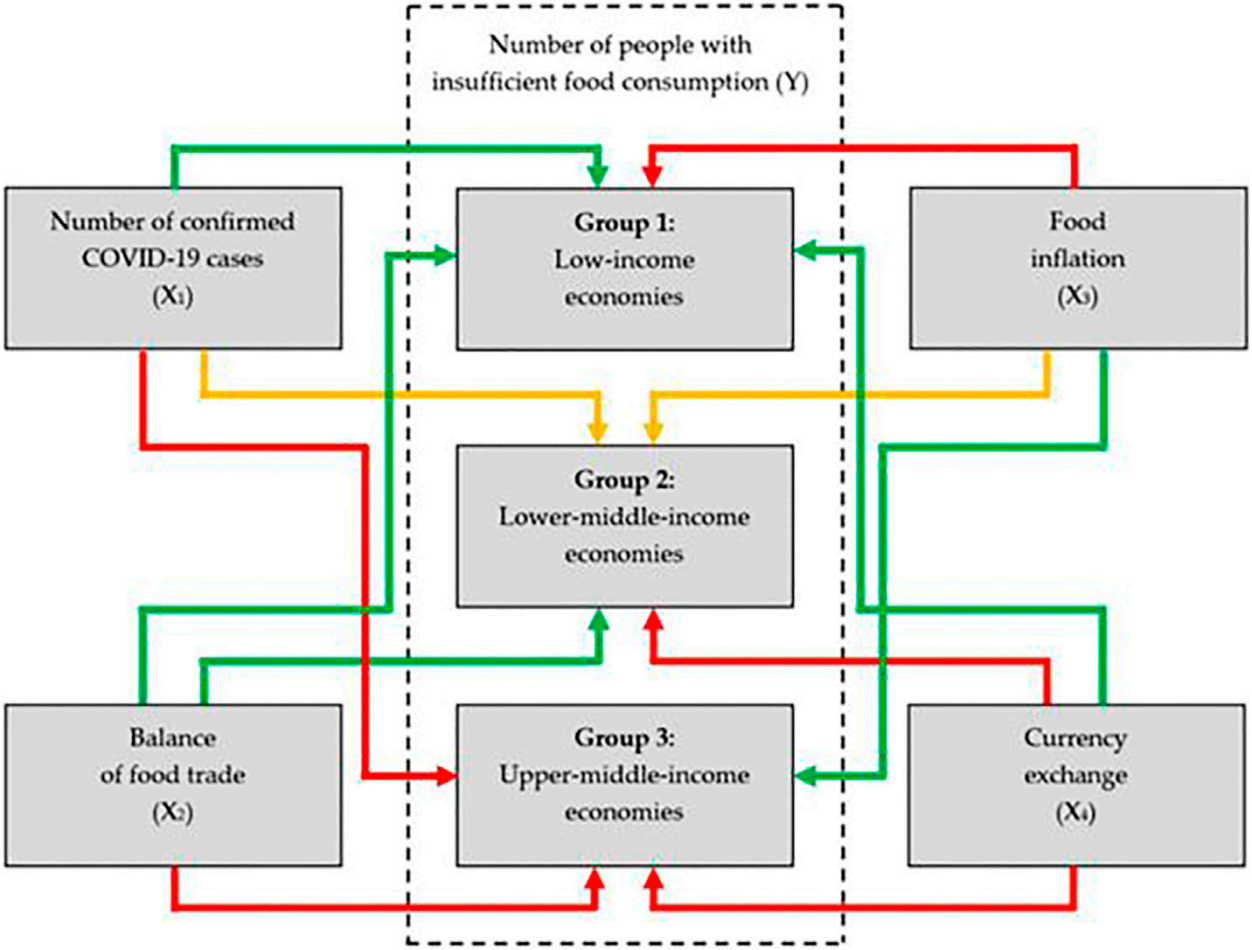
Summary of X (FAO and WHO, 2020; Hamid et al., 2020; IATA, 2020; WHO, 2020a) effects on Y across three groups of countries where Green showing weak influence; yellow shows medium influence and Red showing strong influence (Source: Erokhin and Gao (McMichael, 2004).

Their study explains the cumulative effects of covid-19 pandemic on overall food security in 45 developing nation by including variables X (Hamid et al., 2020; IATA, 2020; FAO and WHO, 2020) as per trends of Hunger map of WFP. The food trade balance (X2) represented the country’s reliance on imports of food and thus revealed improvements in the availability of food. Food inflation (X3) and currency exchange (X4) have been used to demonstrate the effect on food security of changes in access to food and agricultural products (Figure 2). It has been observed that in countries like Ecuador, Pakistan, India, Turkey and Peru (primarily middle-income economies) where the number of reported COVID-19 cases per capita is high, the Y-X1 linkage showed significant results. Lower developing countries are dependent on import for the staple crops as both global food chain disruptions and protectionist trade policies lead to great economic losses could have serious negative consequences for food security [Puma (Wood et al., 2018) and Wood et al. (Frankenberg et al., 2019)].

After evaluating the results of various hypothesis testing, Erokhin and Tianming Gao (Smith and Glauber, 2020) put forth that across Group I, number of people with insufficient food consumption (Y) is unidirectional linked to food trade balance (X2) but the significance of the link is low even in the countries like Tajikistan, Haiti and Guinea where food availability largely depends on imports. For import-dependent upper-middle-income economies, the greatest influence of X2 on Y is seen to be in countries like Algeria, Botswana, Colombia, Jordan, Lebanon. In most low-income nations, a lower proportion of food imports in exchange is correlated with a decline in the percentage of people with inadequate food intake. However, findings of this study concluded that Group I and Group II economies depend on imports less diversified than Group III countries that are more tightly integrated into global supply chains. In the latter situation, greater reliance on imports means that the Food Trade Balance, currency exchange, and therefore the food security position of the people are more affected. In North Africa and the Middle East countries that relies on imports of food and therefore rely on currency fluctuations induced by the pandemic, UNCTAD [95] revealed increased risks for food safety. The best results of X2 and X4 on Y on Algeria and Turkey are seen in favor of this UNCTAD prediction. In another Hypothesis there is an anticipation trend of an increase in the share Y of food trade as well as exchanges in currency, particularly in the countries with the highest middle income. The most significant influence of X2 on Y is expected in Libya, where reliance on food imports exceeds 90% (18.03%). The X2 in Y proportion is almost 12% in March 2021 in Namibia, which is another Category III nation primarily dependent on imports. In countries closely embedded in the global food supply chains, the role of the currency exchange in securing food supply would increase. In Turkey, for example, X4 describes 15.21% of Y. In contrast with those in low income countries, the effect of food inflation on the number of people with inadequate food intake in high-middle income economies has been lower. This result confirms Hypothesis 3 (the effects of X4 and X2 on Y are the highest among the economies included in the study, while that of X3 is the lowest) coincides both with Frankenberg and Thomas (Giordani et al., 2014) and Smith and Glauber (Anderson and Nelgen, 2012) who announce that elevated food price rates have exacerbated poor households’ poverty traps, but have no major impact on the relatively good food safety status. For example, in Cambodia we saw the limited exports of some agricultural produce between March–April 2020, which resulted in the reduction in the number of people with insuccinating food intake, both negative balances of trade in food and low inflation in foodstuffs. Vietnam and Turkey, on the other hand, have not been very active in their decisions to reduce food exports. The ARDL study indicates that a 1% shift in the food trade balance is correlated with a 0.02% rise in food poverty in Vietnam. The X2-Y partnership is poorer but still optimistic in Turkey. This study showed major causal association between X3 and Y in both countries (5%! 0.35% in Turkey and 5%! 0.31% in Vietnam). This finding confirms the estimates of Anderson and Nelgen (Rude and An, 2015), Giordani et al. (Dawe and Peter Timmer, 2012), and Rude and An [68], who found that trade protectionism could cause food inflation and thus intensify food insecurity.

## Conclusion

In the current situation, the global issue is food quality and safety. COVID-19, which provides food coverage for the most vulnerable section of the population at risk, has struck the supply chain the hardest. In general food demand is very inelastic and it takes many years for supply to completely respond to a shift in prices, so the shocks in GDP have only a marginal effect on global production and consumption. The inability to contain the COVID-19 pandemic has had far-reaching consequences for the world economy, with global GDP expected to plummet by 3.3% by 2020. Despite the fact that the global economy is expected to increase by 6% in 2021, recovery will be contingent on fair vaccine distribution worldwide. According to the International Chamber of Commerce, failure to do so might cost the global economy up to $9 trillion, with losses shared evenly by rich and poor countries, wreaking greater economic havoc than the 2008 financial crisis. High value added goods such as meat and milk as well as bio-fuels are the commodities whose production changes the most. In order to ensure the welfare of farm workers, countries should take action. Healthcare workers on staff should monitor employees’ disease status. Countries can create collection centres for agricultural production at locations easily reached by small-scale farmers to minimize mobility. Collection centres for agricultural production should be built to provide high capacity storage for (Beghin, 2014; FAO, 2020a). In order to reduce the depletion of food throughout the food supply chain, enhanced and specialized storage systems should also be used. However when additional capital injection is needed new facilities or improved technology include higher manufacturing costs. Small and medium-sized agricultural firms may also maintain their operations through government or donor capital injections (Anang et al., 2015; World Food Programme, 2020b). Food banks may play a significant role in considering the horizontal and vertical cooperation structures with farmer associations that allow pledged agriculture procedure. Developing countries will suffer the most despite any economic or food crisis mainly because of their limited resources and are subjected to the deterioration of the macroeconomic environment. It is interesting to note that before the pandemic over 2 billion of the most impoverished people in the world spent 70% of their disposable income on food so this reiterates the importance of food security and how a disruption in the supply chain can have serious repercussions on more than a quarter of the world’s population. Unless immediate action is taken, the number of people experiencing acute food insecurity is expected to double to 265 million by 2020, as per the World Food Programme (2020b). However, even in Developed nations, more vulnerable groups such as the elderly, chronically ill, and poorer households may be at danger, and COVID-19 has exposed pre-existing social protection deficiencies (OECD, 2020b). Future comparative sociological research on food safety can be built on recent research which has established sociological positions on this arena. It is traditionally dominated by research that overly concerns production and supply and is not sufficiently concerned with the conflicts, stratification and inequality most essential to starvation. The European Union’s Farm to Fork Strategy described food system conversion system to focus on future resilience, health and sustainability. Their issued statement regarding framework were focused on that there can be environmental, health and social benefits, economic gains and ensuring that a resurgence from the crisis can lead us to the sustainable route (European Commission, 2020b).
